# Minimization of Welding Distortion in Large-Scale Welded Structures Using the MAG Method: Numerical and Physical Simulation of the Welding Process

**DOI:** 10.3390/ma19163530

**Published:** 2026-08-20

**Authors:** Marek Mróz, Robert Czech, Janusz Pikuła, Marcin Spólnik

**Affiliations:** 1 Department of Foundry and Welding, Rzeszow University of Technology, ul. Żwirki i Wigury 2, 35-036 Rzeszów, Poland; 2 Naftoremont-Naftobudowa Sp. z o.o., Branch Jedlicze, ul. Sikorskiego 17, 38-460 Jedlicze, Poland; 3 Łukasiewicz—Upper Silesian Institute of Technology, Centre of Welding, ul. K. Miarki 12-14, 44-100 Gliwice, Poland; janusz.pikula@git.lukasiewicz.gov.pl

**Keywords:** MAG welding, large-scale storage tank bottom, welding distortion, numerical simulation, physical simulation

## Abstract

This paper presents the results of research on minimizing welding distortion in a large-scale storage tank bottom made of S235JR steel with a diameter of 10,000 mm. The welding technology previously employed guaranteed flatness tolerances of the bottom plates in accordance with the EN 14015 standard at a ratio of T = 0.25% × D = 25 mm, where D represents the diameter of the tank bottom. The objective of this study was to investigate the possibility of reducing this value to the target level of ΔT = 0.2% × D = 20 mm. Two clamping system configurations, used to restrain the tank bottom plates during welding, were analyzed. Numerical simulations of both configurations were performed using the finite element method (FEM) in the SYSWELD software. For the selected clamping configuration, a physical simulation of the welding process was carried out, followed by an analysis of the distribution of welding-induced distortions in the storage tank bottom with respect to compliance with the required bottom plate flatness tolerance of 0.2% × D.

## 1. Introduction

Welding residual stresses and their associated distortions represent one of the most critical challenges in the fabrication of welded structures, particularly those characterized by large scales and complex geometries.

A common feature of all welding methods is the utilization of a heat source to melt the filler metal and form a molten weld pool, which subsequently solidifies into a weld bead during rapid cooling. This heat source also affects the surrounding regions of the welded joint, specifically the base metal zones immediately adjacent to the weld bead, known as the heat-affected zone (HAZ).

Factors occurring during the thermal cycle of the welding process—such as rapid heating and cooling, degradation of mechanical properties, and phase transformations in the HAZ—result in mechanical interactions between adjacent areas of the welded structure. These regions experience high temperature gradients, thereby generating the internal stresses that drive welding distortion. During joint formation, the solidification process induces compressive stresses within the weld bead, while tensile stresses develop within the HAZ. When these stresses exceed the yield strength of the material, permanent deformation of the welded joint occurs.

For steel, the primary parameters governing mechanical behavior include the longitudinal modulus of elasticity (E), the shear modulus (G), Poisson’s ratio, yield strength (R_e_), and the coefficient of linear thermal expansion. The temperature dependence of these properties is well established. Specifically, the values of the E and G moduli, as well as the yield strength, decrease with increasing temperature, consequently increasing the susceptibility to plastic deformation (welding distortion). Conversely, the coefficient of linear thermal expansion increases with temperature, further contributing to the propensity for welding distortion.

While the total elimination of post-weld residual stresses is difficult to achieve, mitigating their impact on the quality of the welded structure is essential. Welding engineers can utilize a variety of approaches to accomplish this objective, including both thermal and mechanical methods. Furthermore, minimizing the impact of factors that induce welding distortion can be achieved through the optimal selection of welding process parameters. These activities are frequently associated with increased financial expenditures and extended production lead times [1,2].

One approach to reducing welding stresses and distortion is to limit the heat input during the welding process. Currently, methods utilizing variable-polarity pulsed current, as well as low-energy consumable electrode welding processes such as AC Pulse, Cold Process, and CMT Advanced, are increasingly being adopted [3,4,5,6].

Designers of welded structures and welding engineers have a variety of approaches, methods, and tools at their disposal to mitigate the occurrence of welding stresses. These include:

- Designing structures with a minimal number of welded joints (welds);
- Limiting the cross-sectional area and length of the welds;
- Utilizing intermittent welds;
- Employing double-sided butt welds;
- Avoiding intersecting welds;
- Implementing appropriate preparation methodologies for the components to be joined;
- Selecting suitable welding methods and bead deposition techniques;
- Utilizing backing bars or components with alternative geometries for forced cooling of the welded joint during welding;
- Applying elastic presetting;
- Mechanical stress relieving (pre-stiffening).

When designing welded structures, a minimal number of welds should be pursued by utilizing hot-rolled or cold-formed structural sections (such as angles, tees, and channels) as structural components. Concurrently, the positioning of the welds along or near the neutral axes of these structural sections must be taken into account. Excessive total heat input in the welding process, resulting from a high volume of deposited welds, can be decreased by limiting both the length and cross-sectional area of the welds, provided that the required mechanical strength properties of the welded joint are maintained. The length of the welds can be restricted through the use of rolled structural sections. Reducing the weld cross section can be achieved by employing partial penetration butt welds. Measurable reductions in the thermal effects of the welding cycle are provided by the use of intermittent welds. However, it must be noted that this solution cannot be implemented for welded structures operating under cyclic loading conditions or within aggressive corrosive environments. Transitioning the butt weld configuration from single-sided to double-sided facilitates a reduction in welding distortion. This is linked to the influence of the appropriate deposition sequence for individual weld beads. Sectioning the structure into distinct components that are subsequently joined (welded) represents an effective method for reducing welding distortion. This approach should be utilized by structural designers during the design phase, particularly to avoid intersecting welds. Furthermore, the weld bead deposition technique significantly affects the minimization of welding distortion. Welding in segments using alternating sequences or the backstep method results in a substantial reduction in welding distortion. A frequently utilized approach for mitigating welding distortion is the application of elastic presetting. Proper selection of the presetting magnitude ensures that the fabricated welded structure achieves the required shape and specified dimensions. The magnitudes of these pre-deformations depend on the welding method and the bead deposition technique.

For large and complex welded structures characterized by a diverse combination of short and long welding thermal cycles, the application of mechanical stress relieving (pre-stiffening) is an effective method to ensure the minimization of welding distortion. By applying an appropriate external load to the welded structure, a reduction in residual stresses is achieved. To accomplish this, it is necessary to satisfy the condition where the sum of the stresses originating from the external load and the residual stresses equals the yield strength of the welded material.

Research on welding distortion can be conducted either by executing an actual welding process (physical simulation) or by utilizing numerical simulations via the finite element method (FEM). In many industrial fields, the design process is increasingly supported by FEM analyses [7,8]. This allows for the verification of the designed model’s integrity prior to its actual fabrication. Numerical simulation is primarily applied to determine the temperature fields generated during welding, as well as the resulting stresses and distortions of the welded components. Based on the calculated temperatures during the welding process, continuous cooling transformation diagrams, and material hardness data, it is possible to determine the structural phases and hardness of the material subjected to the welding thermal cycles. The utilization of numerical methods, particularly during the initial stage of developing the welding technology and sequence plan, enables a reduction in the number of trials required to establish the optimal welding process parameters. Furthermore, it eliminates design-phase errors that are highly difficult or virtually impossible to rectify during subsequent production stages. An additional advantage of implementing numerical methods is the capability to simulate the welding processes of larger sections or even the entire structures to be welded. Relative analyses and optimization of the welding process make it possible to evaluate numerous fabrication variants for welded structures without the need to perform physical trials. The capability to perform physical simulations prior to implementing technology into production, particularly for large-scale structures, is restricted primarily due to the associated costs and time requirements.

Welding distortions are also detrimental to the load-bearing capacity of a welded structure, as they can superimpose onto stresses induced by external loads. Such a stress state can lead to a reduction in the safe fatigue life of the structure or cause brittle fractures during the initial stages of operation. In butt joints, the primary issue involves angular distortions caused by transverse shrinkage, which manifest as component deflection. In the case of thin plates, buckling due to longitudinal shrinkage is also characteristic. Mitigating these discontinuities is achievable through appropriate technical solutions, such as the proper design of the structure and material selection, partitioning the structure into subassemblies, or applying fixturing, which limits the effects of longitudinal shrinkage when welding thin plates [8].

The development of computer simulation techniques has led to welding process simulations being increasingly utilized to optimize and improve the manufacturing technology of welded structures. Employing advanced software to numerically simulate the phenomena and processes occurring during welding allows for a reduction in both the development time of manufacturing technologies for welded structures and financial expenditures [9,10]. Numerical simulations are utilized in welding technologies such as MIG [1,11,12,13,14,15,16], MAG [12,14,17,18,19], TIG [9,20,21,22,23], NG-TIG [12], TIG-MIG [24], and EBW [12]. Various software packages, primarily based on the finite element method (FEM), are used for the numerical simulation of the welding process. These include programs such as ABAQUS [1,2,9,23,25], ANSYS [13,15,17,20,21,26], and SYSWELD [1,12,22,27].

Due to the large volume of complex computations, the numerical simulation of welding processes requires high-performance computing power. For this reason, various simplifications are applied to reduce execution time, such as utilizing axisymmetric elements or replacing 3D models with simplified 2D planar elements [1,10,18,27].

The numerical simulation of a welding process should ensure results that correspond as closely as possible to the actual welding process (physical simulation). However, as emphasized by the authors of [13,23], this requires accounting for numerous variables describing the heat source, the welding method and its technological parameters, the joint geometry, the bead deposition technique, and the quantity and configuration of the fixture (stiffening) system. This is particularly problematic in the case of large-scale welded structures. The authors of [2,19], when analyzing the welding deformations and distortions of large-scale welded structures, observed high correlation between the numerical simulation results and those obtained under actual welding process conditions (physical simulation). Conversely, the authors of [28] presented the results of numerical analyses regarding the welding of storage tanks. They highlighted the challenges associated with applying FEM to determine the welding distortion of large-scale structures.

As asserted by the authors of [29,30,31,32], the feasibility of conducting accurate thermo-mechanical FEM analyses is restricted to small-scale components. Modeling large structures entails extensive computational time. To reduce this execution time, solutions are employed that involve applying a specified welding thermal cycle to sequentially designated segments of the joint.

Predicting component mismatches during assembly and mitigating them as early as the design and assembly planning phases enables a reduction in costs and delays arising from distortion rectification. In numerous industrial sectors, the design process is increasingly supported by FEM analyses, which facilitates the verification of the designed model’s integrity prior to its fabrication [33,34,35,36,37,38].

Modeling and executing computations for large structures is inherently associated with prolonged computational times. To address this, solutions are implemented that enable sufficiently accurate results to be achieved in a significantly shorter duration. Generally, these methods involve applying a specific welding thermal cycle over designated segments of the joint rather than along its entire length. Another solution is the implementation of the shrinkage method, which involves applying deformations within the joint zone that correspond to the cooling phase of the joint following the welding process [30,32,35,36,37].

The literature indicates that fully coupled thermo-elastoplastic analyses provide the highest level of accuracy in reproducing the welding process. However, their application to large-scale structures is often significantly limited by the very high computational requirements involved. For this reason, inherent strain-based methods are widely employed for the analysis of large-scale structures, enabling a substantial reduction in computational time while maintaining satisfactory agreement with experimental results [39,40,41,42].

Among the main advantages of the shrinkage method is its ability to efficiently investigate the effects of different technological variants, welding sequences, restraint conditions, and structural configurations with relatively low computational effort. However, it should be emphasized that this method constitutes a significant simplification of the actual welding process. It does not directly reproduce local temperature fields, heat transfer phenomena, temperature-dependent changes in material properties, phase transformations, or the actual evolution of residual stresses. Consequently, the results obtained are highly sensitive to the manner in which equivalent strains are defined and to the modeling assumptions adopted. The literature indicates that the accuracy of quantitative deformation predictions depends to a large extent on the method used to determine the equivalent strain parameters and on the quality of their calibration. The resulting deformation level is influenced, among other factors, by joint geometry, welding heat input per unit length, material thickness, structural restraint conditions, and the procedure used to determine shrinkage strains for individual joint types. In the absence of adequate calibration, the predicted deformation values may deviate from experimental results, even when the overall deformation pattern of the structure is correctly reproduced. Therefore, the shrinkage method represents an effective tool for supporting the analysis and optimization of welding technologies for large-scale structures. At the same time, achieving a high level of quantitative agreement between numerical predictions and experimental measurements requires careful definition of the modeling assumptions and appropriate calibration of the shrinkage parameters for the joints under consideration, based on experimental data or previously validated numerical models [41,42].

The objective of this study was to optimize the welding technology for a large-scale storage tank bottom made of S235JR steel using numerical simulation (FEM). The welding technology previously employed guaranteed flatness tolerances of the bottom plates in accordance with the EN 14015:2004 standard [43] (Specification for the design and manufacture of site built, vertical, cylindrical, flat-bottomed, above ground, welded, steel tanks for the storage of liquids at ambient temperature and above) at a distortion-to-diameter ratio of ΔT = 0.25% × D = 25 mm, where D represents the diameter of the tank bottom. To reduce the tolerance ratio to a level of ΔT = 0.2% × D = 20 mm, two variants of a fixture system—designed to stiffen the tank bottom during the welding process—were developed, for which numerical simulations were performed within the SYSWELD software ver.16 environment. For the selected fixture system configuration, a physical simulation of the welding process was conducted, and the resulting welding distortion distribution across the storage tank bottom was analyzed regarding compliance with the 0.2%x*D plate flatness tolerance.

## 2. Methods, Results and Discussion

**Figure 1:**
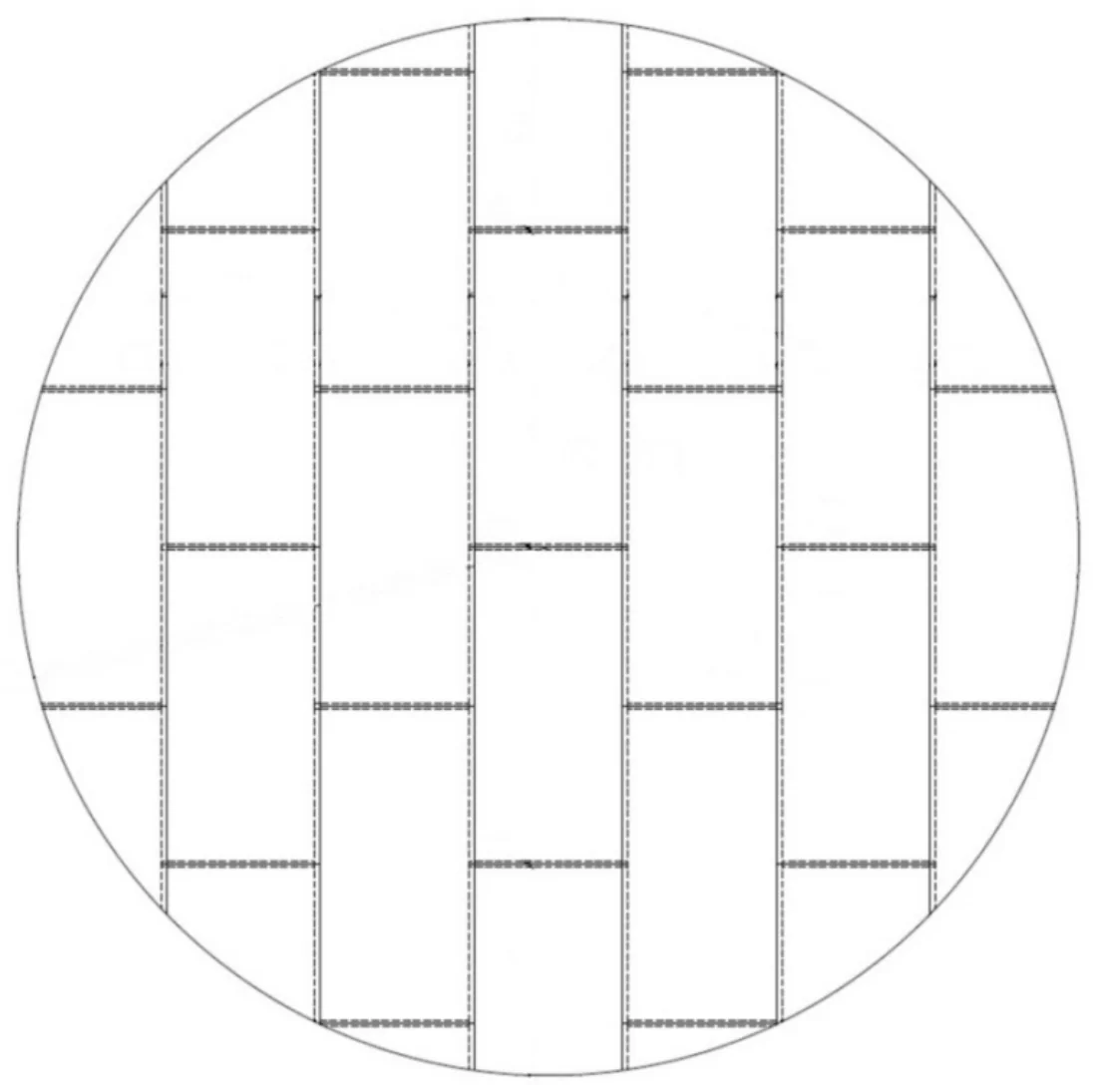
Storage tank bottom with a diameter of 10,000 mm.

The subject of this study was a storage tank bottom with a diameter of 10,000 mm, fabricated from S235JR steel plate elements with a thickness of 6 mm. The welding process was executed using the MAG method. The bottom structure incorporates both butt and fillet joints. A schematic diagram illustrating the arrangement of the welded joints on the large-scale storage tank bottom is presented in Figure 1.

### 2.1. Numerical Simulation

#### 2.1.1. Numerical Simulation Methodology

**Figure 2:**
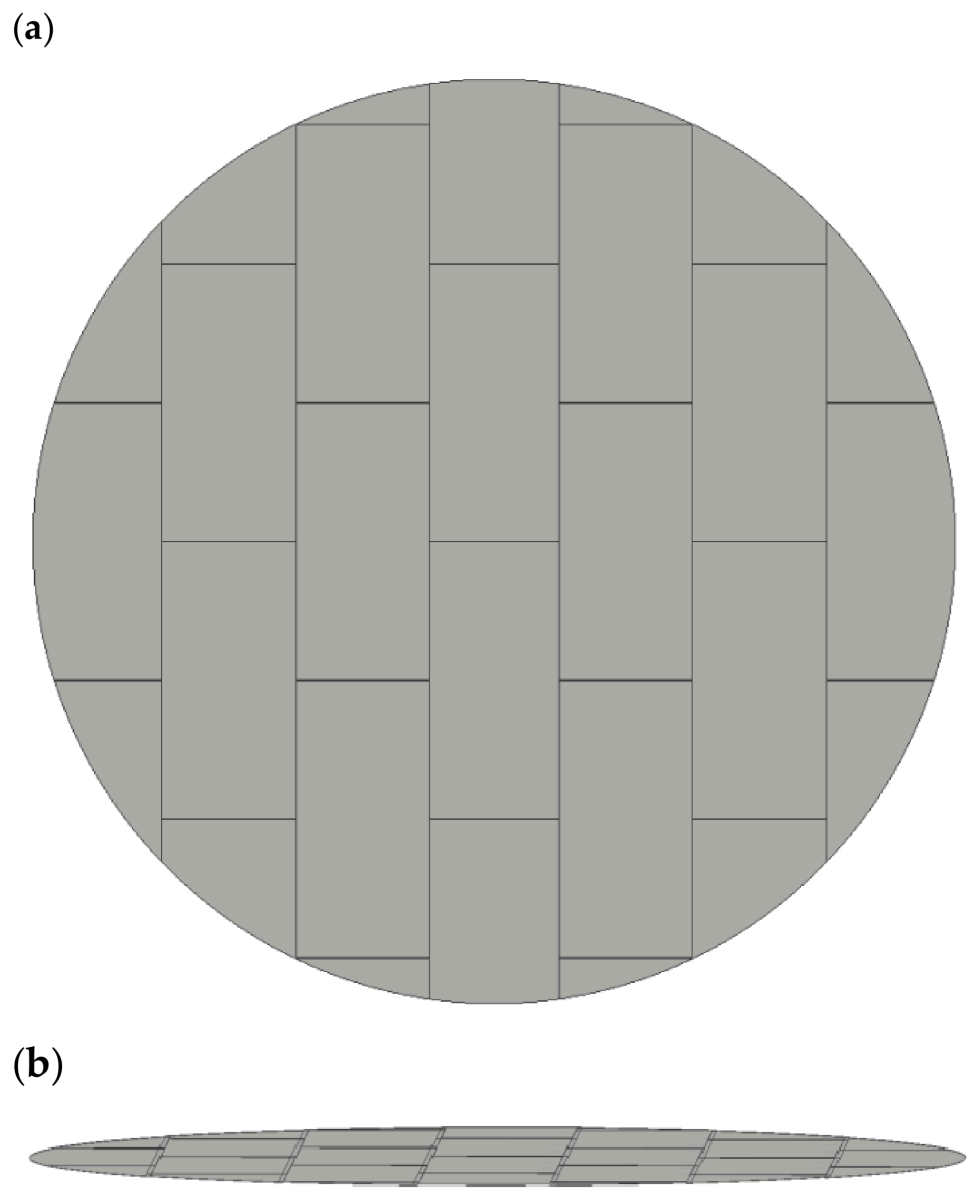
Geometry of the storage tank bottom: (**a**) top view, (**b**) side view.

**Figure 3:**
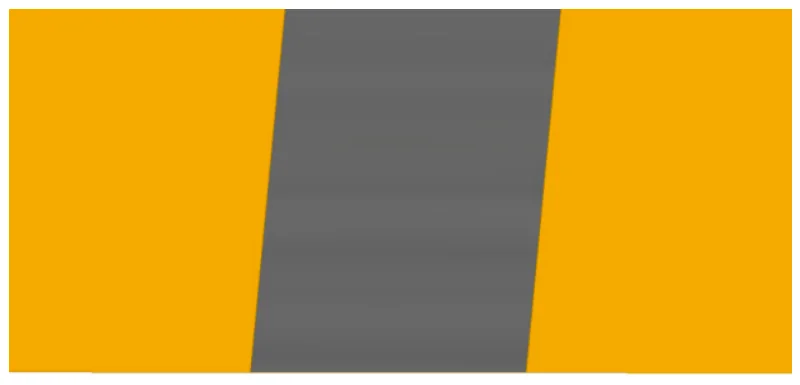
View of the butt welds modeled using mid-surfaces.

**Figure 4:**
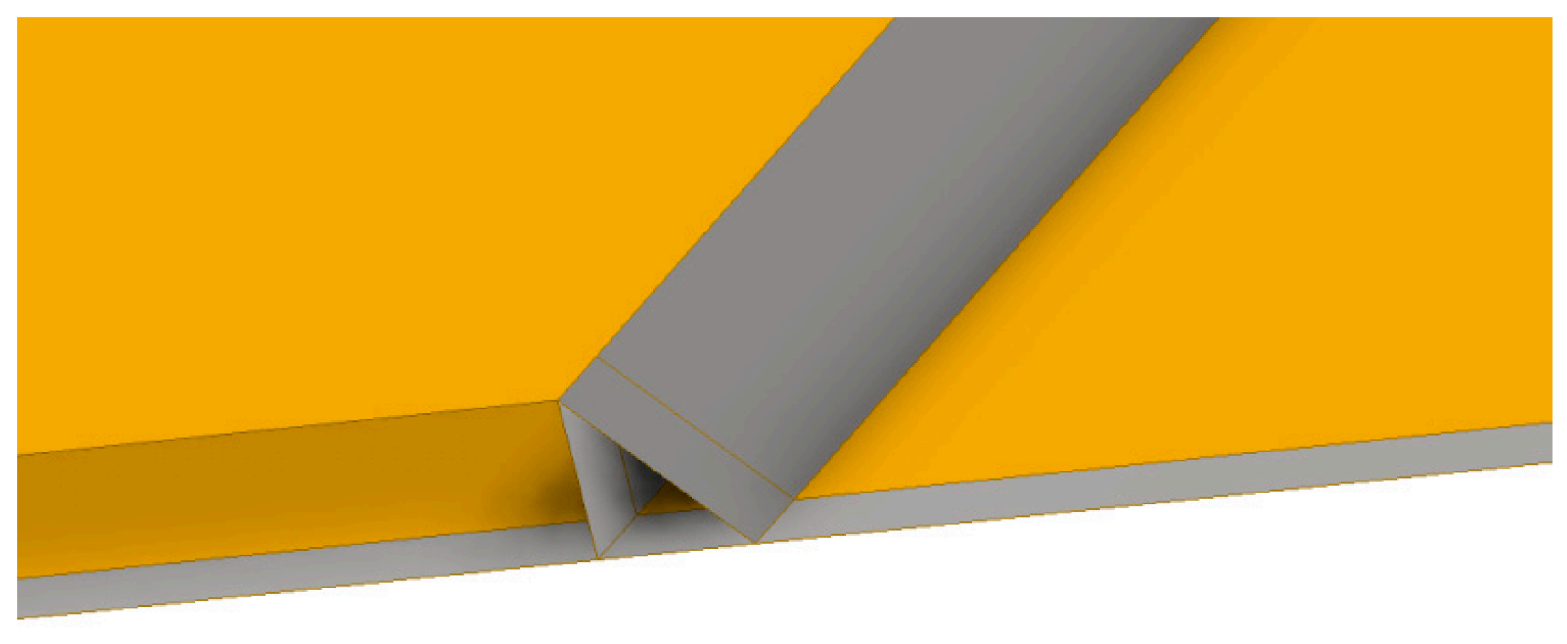
View of the lap joints modeled using weld face surfaces.

The initial phase of the numerical simulation involved the preparation of CAD and FEM models of the tank bottom mockup (Figure 2). For the CAD model, a geometry consisting of mid-surfaces was generated first. The subsequent stage of CAD model development involved creating the butt and fillet welds. The butt welds were modeled using mid-surfaces (Figure 3), whereas the fillet welds were represented using the weld face surfaces (Figure 4).

The width of the butt welds was determined based on the welding groove geometry defined in the Welding Procedure Specification (WPS).

The numerical simulation was performed using SYSWELD 16 software. In modeling the execution of the welded joints, the shrinkage method was applied—a technique based on imposing shrinkage within the joint zones, the magnitude of which is defined by the thermal expansion coefficient of the material. In this method, the weld segment being deposited at a given moment is defined, and a condition inducing shrinkage in that specific region is prescribed.

The shrinkage method represents a simplified approach to simulating welding distortion. This approach serves as an alternative to fully coupled transient thermo-mechanical analyses and is based on the concept of inherent strain, which enables the reproduction of the global effects of the welding process without the necessity of detailed transient temperature field modeling [39,40].

In classical mechanics of materials, the total strain of a material can be expressed as the sum of its elastic, plastic, and thermal components:

*ε* = *ε*_S_ + *ε_p_* + *ε_th_* (1)

where *ε* is the total strain, *ε*_s_ is the elastic strain, *ε_p_* is the plastic strain, and *ε_th_* is the thermal strain.

The thermal strain depends on the coefficient of thermal expansion of the material and the temperature increment:

*ε_th_* = α · ΔT (2)

where *ε_th_* is the thermal strain, α is the coefficient of linear thermal expansion, and ΔT is the temperature increment (difference).

Accurate determination of all strain components for actual welding processes requires a complex thermo-elasto-plastic analysis that accounts for the local temperature field, non-linear material properties, and structural fixturing conditions [44]. Furthermore, describing the temperature field during the welding process may require advanced heat conduction models that extend beyond the classical Fourier law. In particular, dual-phase lag models allow for the incorporation of delays in heat flux propagation, although they significantly increase the complexity of numerical computations [45,46]. Due to the aforementioned limitations, engineering analyses utilize simplified methods in which the effects of the welding process are represented by imposing effective shrinkage strains within the weld zones. In the shrinkage method, these strains represent the averaged effect of thermal and plastic interactions occurring during the welding thermal cycle [39,41,47].

Formalized mathematically, the shrinkage strains can be expressed as:

ε_sh_ = β · α · ΔT (3)

where ε_sh_ is the shrinkage strain, β is a coefficient accounting for the influence of plastic processes and structural displacement constraints, α is the coefficient of linear thermal expansion, and ΔT is the temperature difference.

Within the context of the finite element method (FEM), the shrinkage method permits the utilization of the equivalent load method derived from the assumed inherent strains. In practice, shrinkage strains can be implemented directly within the elements corresponding to the weld or via equivalent nodal forces that generate the appropriate stress and displacement fields [48,49].

The relationship between strains and stresses in the material within the elastic range is governed by Hooke’s law, meaning that the prescribed shrinkage strains induce a residual stress field and corresponding structural distortions.

The application of simplified computational models is widely established in computational mechanics, particularly in the analysis of complex structures. The finite element method enables the efficient determination of stress and strain distributions within acceptable computational times, which is of paramount importance in the optimization of structures and manufacturing technologies [50,51].

Regarding the analysis of welding processes, numerical methods constitute a vital tool supporting the selection of technological parameters and the mitigation of structural distortions. Although FEM-based simulations allow for the analysis of coupled thermal and mechanical phenomena, engineering practice frequently favors simplified approaches that enable the analysis of large structures while substantially reducing computational time [52,53]. Consequently, the shrinkage method is particularly advantageous in the analysis of large-scale structures where the determination of global distortions is critical and a detailed reproduction of the local transient welding process is not required [42]. However, it must be emphasized that this approach does not directly account for temperature distributions, phase transformation kinetics, or local heat source interaction effects. Therefore, the obtained results should be interpreted as an approximate evaluation of the influence of technological variants on structural distortion rather than an exact representation of the physical welding process history [42,54].

**Figure 5:**
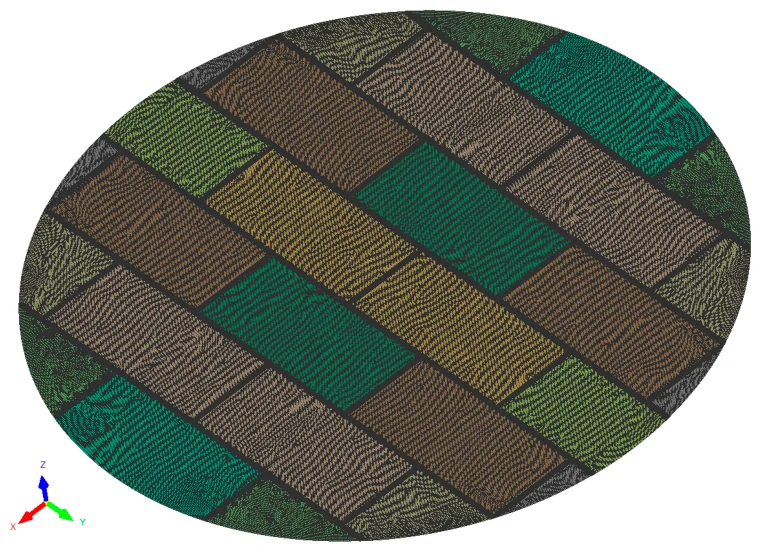
Finite element mesh of the tank bottom model.

Based on the CAD model, a finite element mesh was generated (Figure 5). This mesh was constructed using second-order shell elements (with a quadratic shape function) at an appropriate density according to FEM rules. In critical areas (the welded joints), the mesh was refined. The final FEM model consisted of 432,000 elements and 1,269,000 nodes.

**Figure 6:**
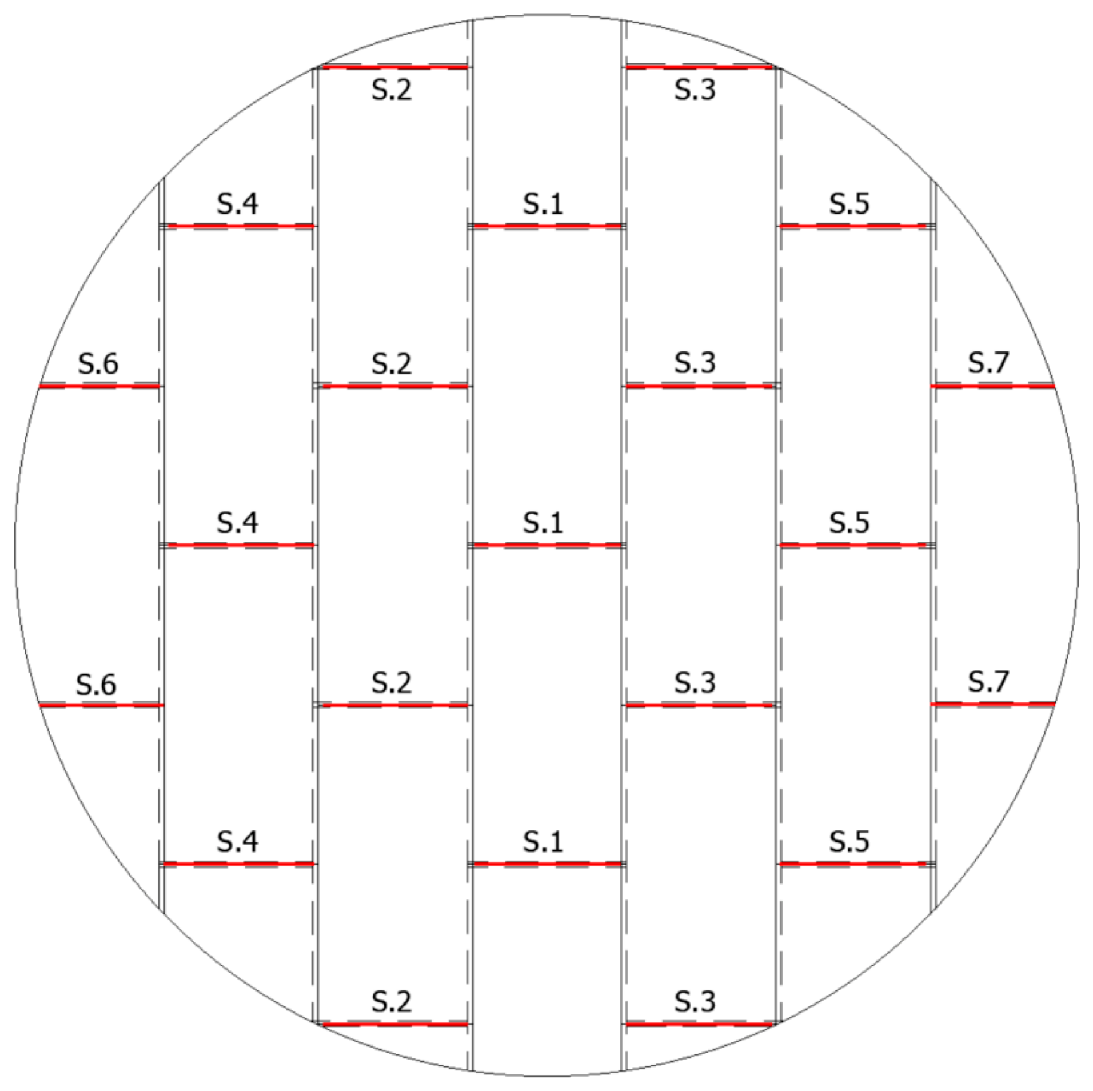
Stage 1 of deposition for butt welds S.1–S.7.

**Figure 7:**
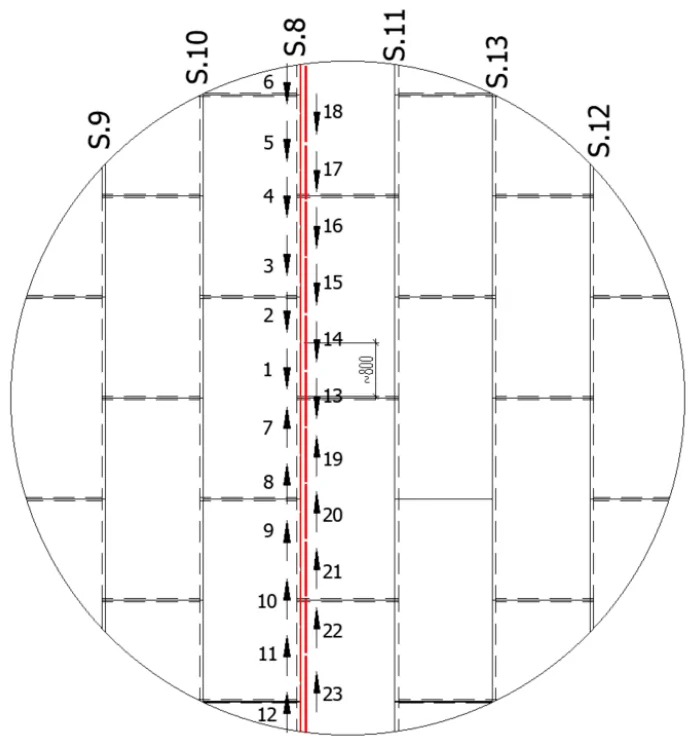
Stage 2 of deposition for fillet welds S.8–S.13.

The numerical analysis was performed for the following sequential welding stages:

- Butt welds S.1–S.7 (Figure 6);

- Fillet welds S.8–S.13 deposited in two passes (Figure 7).

**Figure 8:**
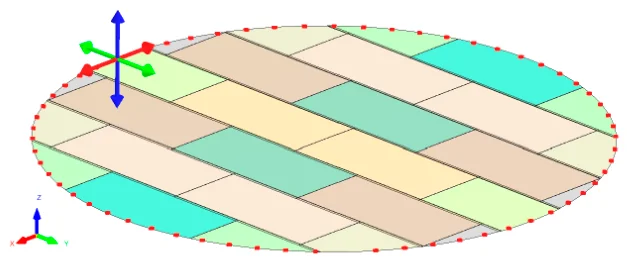
Arrangement schematic of fixtures (indicated in red) during welding in Variant 1; restrained degrees of freedom in the X, Y, and Z directions.

**Figure 9:**
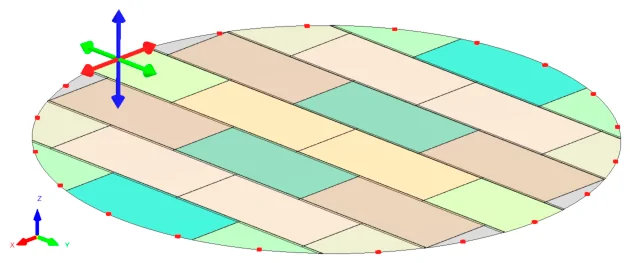
Arrangement schematic of fixtures (indicated in red) during welding in Variant 2; restrained degrees of freedom in the X, Y, and Z directions.

**Figure 10:**
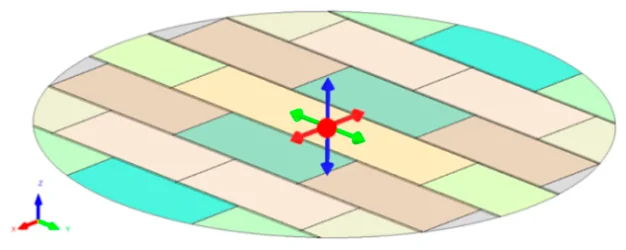
Modification of boundary conditions after releasing the component from the fixturing—restrained degrees of freedom in the X, Y, and Z axes directions.

The numerical FEM simulation was conducted for two variants of fixturing, representing the structural constraints applied to the welded components (Figure 8 and Figure 9). The second model utilized three times fewer fixture points compared to the first variant. The release of the welded structure from its fixtures upon completion of welding was modeled for each variant by transitioning the boundary conditions shown in Figure 8 and Figure 9 to those specified in Figure 10.

**Figure 11:**
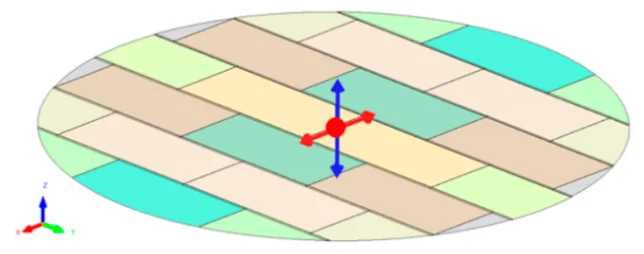
Modification of boundary conditions after releasing the component from the fixturing—restrained degrees of freedom in the X and Z axes directions.

**Figure 12:**
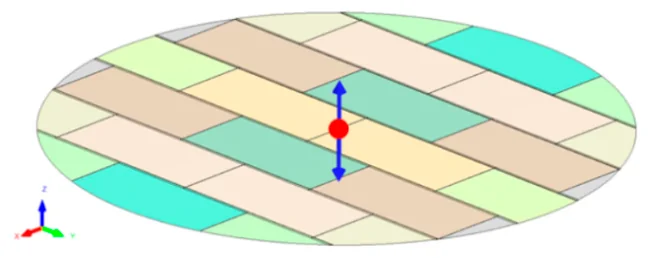
Modification of boundary conditions after releasing the component from the fixturing—restrained degrees of freedom in the Z axis direction.

The modification of these conditions involved restoring the nodes to their pre-welding state positions:

- Fixture boundary condition–zero displacement permitted for the node in the X, Y, and Z axes directions (Figure 10);

- Fixture boundary condition—zero displacement permitted for the node in the X and Z axes directions (Figure 11);

- Fixture boundary condition—zero displacement permitted for the node in the Z axis direction (Figure 12).

#### 2.1.2. Numerical Simulation Results and Analysis

**Figure 13:**
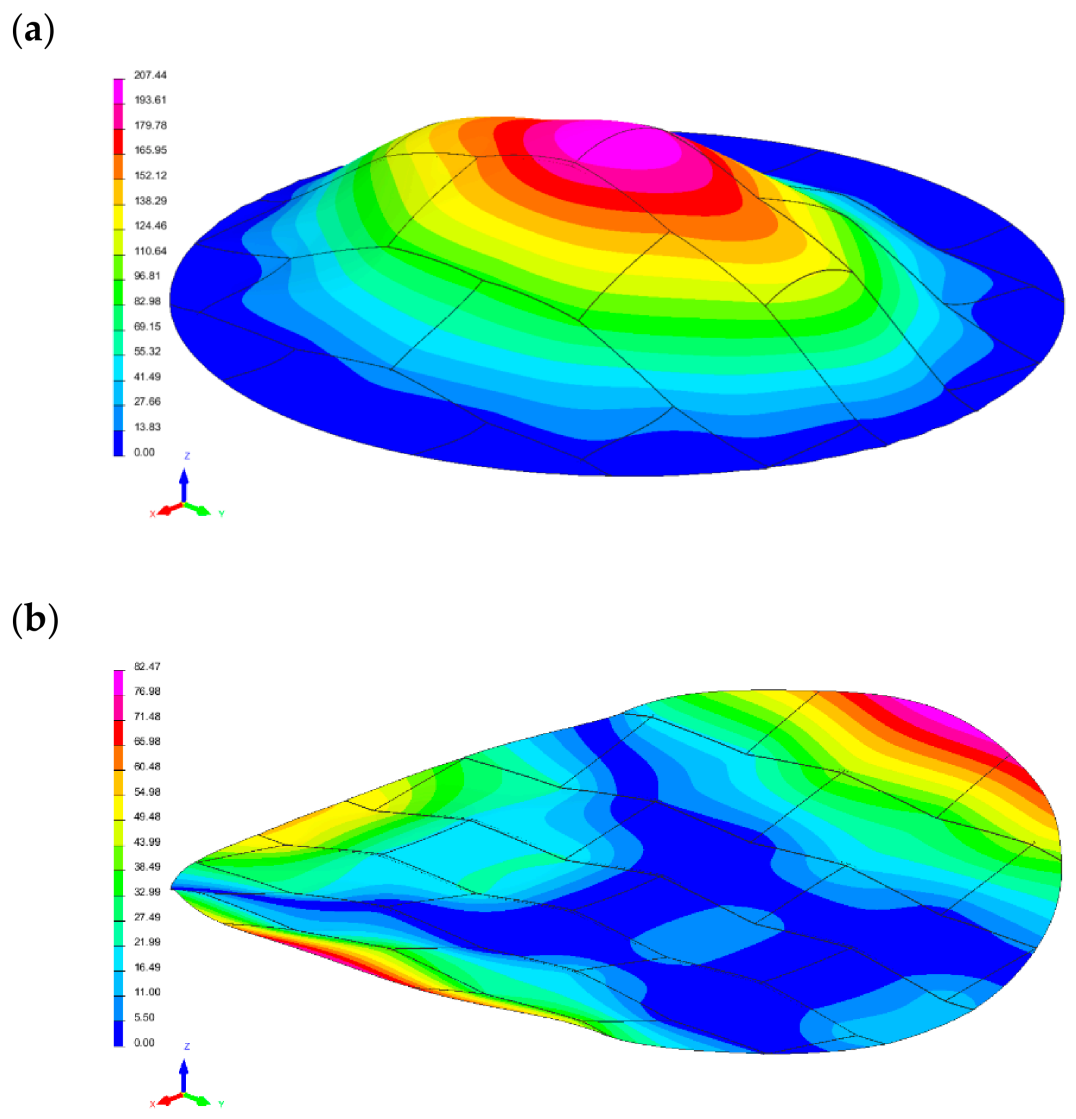
Resultant distortion distribution in the first stiffening variant: (**a**) upon completion of the welding process before fixture release; (**b**) upon completion of the welding process and after fixture release. Component deformation in mm, scaled 10-fold.

**Figure 14:**
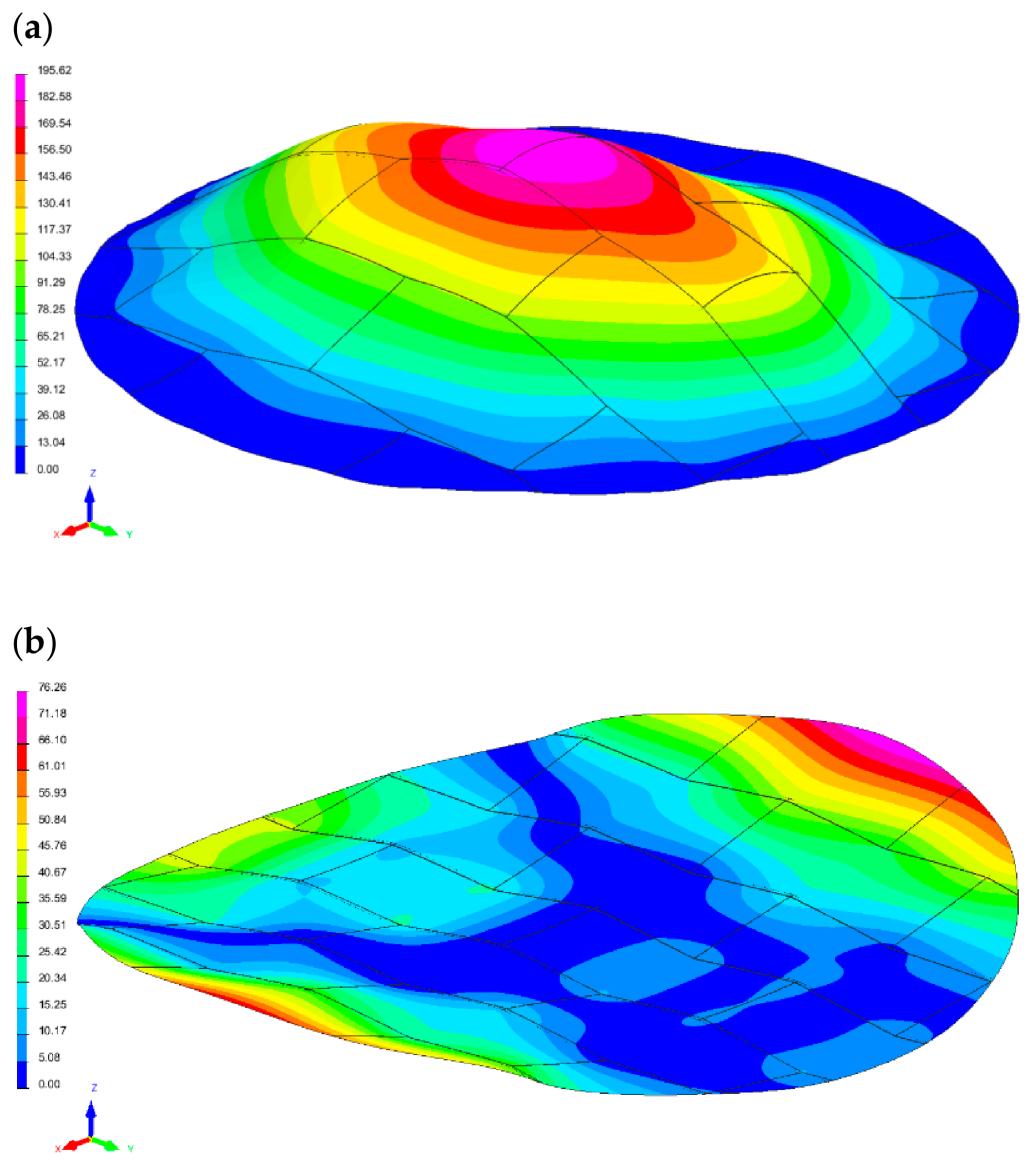
Resultant distortion distribution in the second stiffening variant: (**a**) upon completion of the welding process before fixture release, (**b**) upon completion of the welding process and after fixture release. Component deformation in mm, scaled 10-fold.

The resultant distortion distributions before and after releasing the structure from the fixtures are presented in Figure 13 and Figure 14. For both fixturing variants, the distortion fields exhibit a similar character, with minor variations in distortion magnitudes. In Variant 1, the distortion values prior to fixture release are 6% higher than those in Variant 2, and 8% higher during the stage following release from the stiffeners. The maximum displacements occurred along the Z axis.

**Figure 15:**
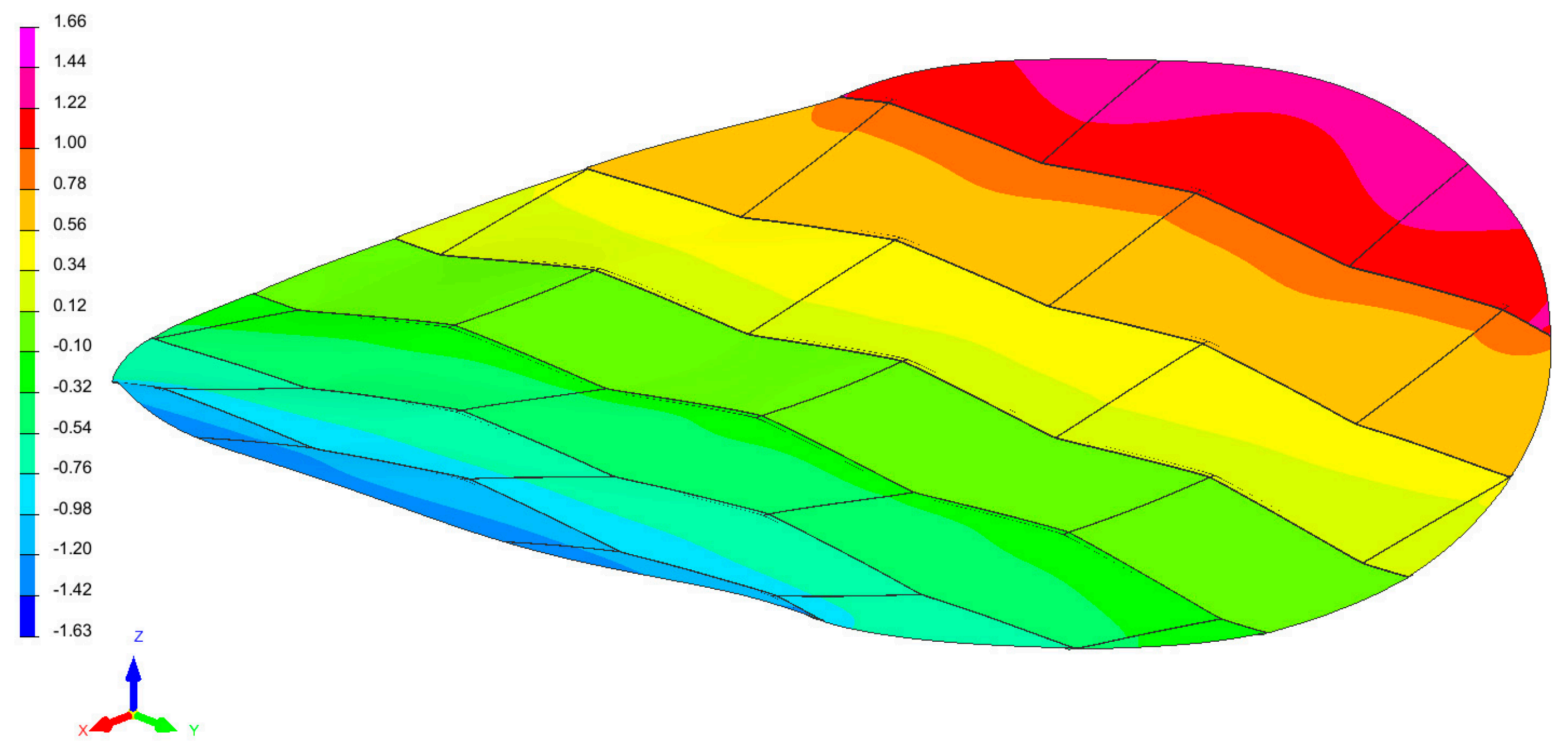
Absolute distortion distribution along the X axis in the first fixturing variant. Component deformation in mm, scaled 10-fold.

**Figure 16:**
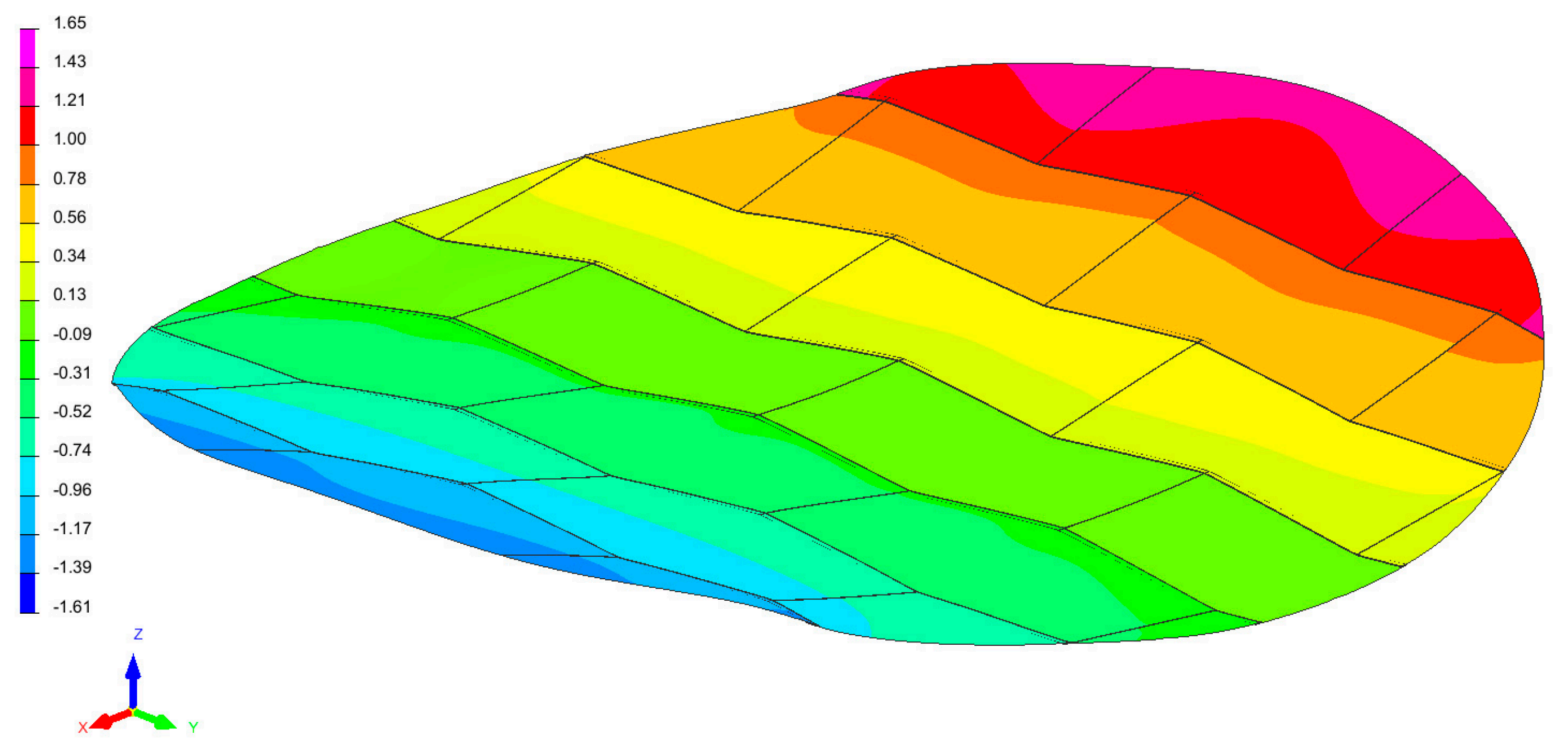
Absolute distortion distribution along the X axis in the second fixturing variant. Component deformation in mm, scaled 10-fold.

**Figure 17:**
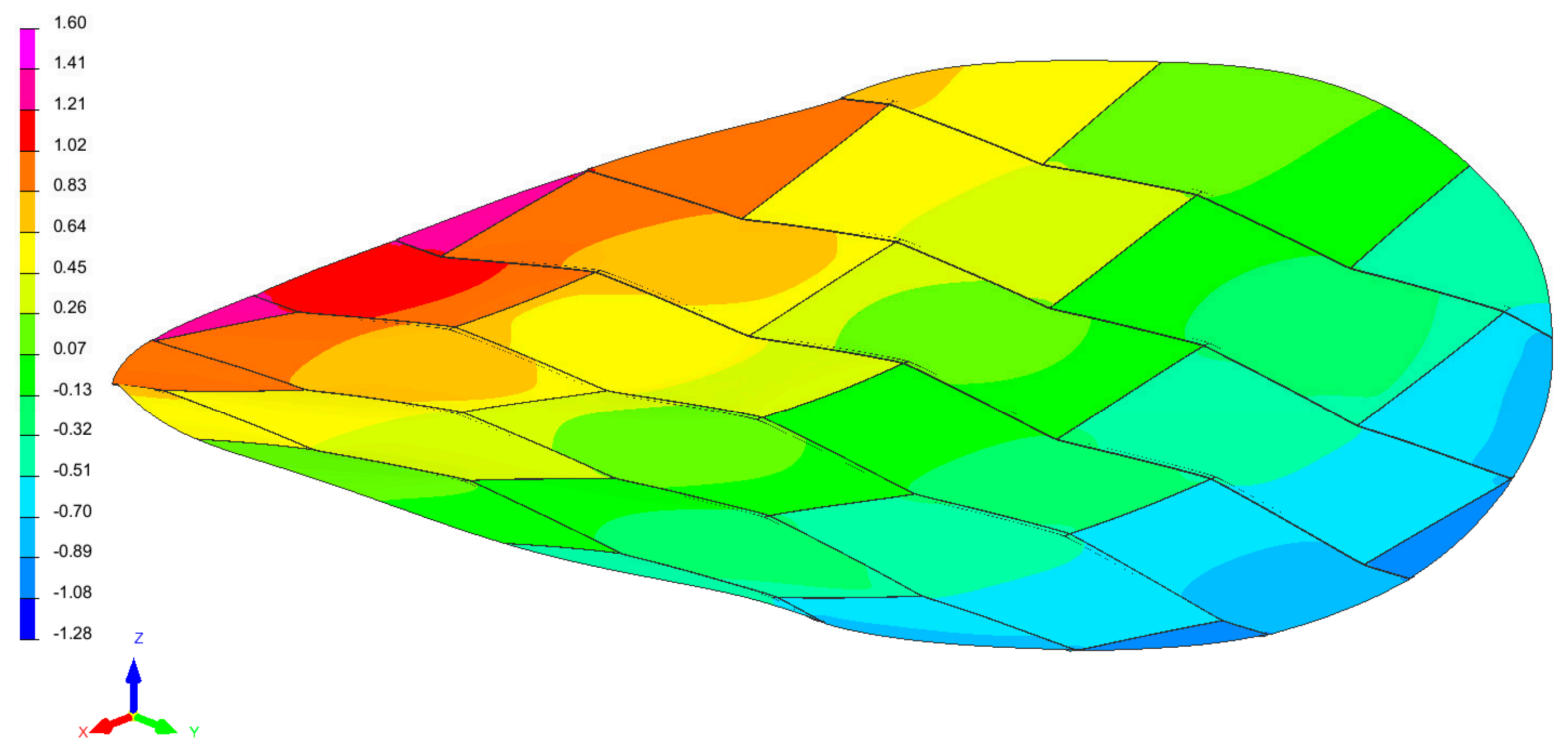
Absolute distortion distribution along the Y axis in the first fixturing variant. Component deformation in mm, scaled 10-fold.

**Figure 18:**
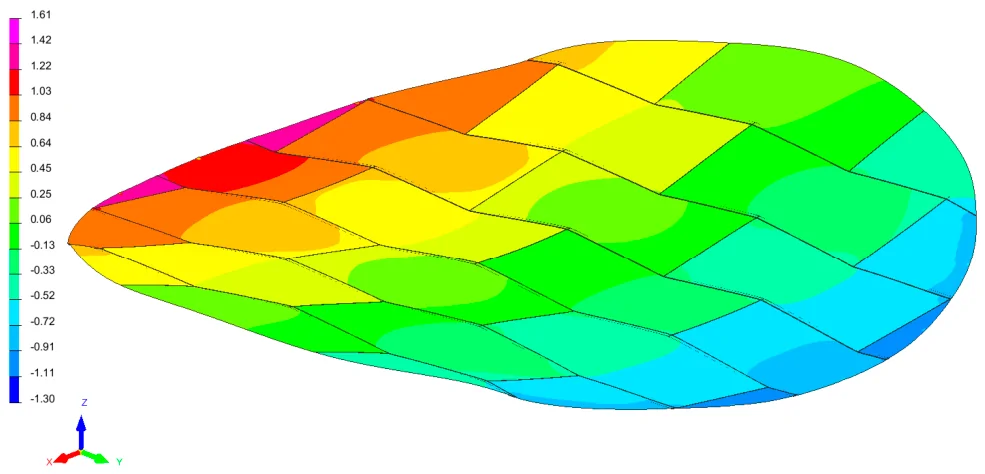
Absolute distortion distribution along the Y axis in the second fixturing variant. Component deformation in mm, scaled 10-fold.

**Figure 19:**
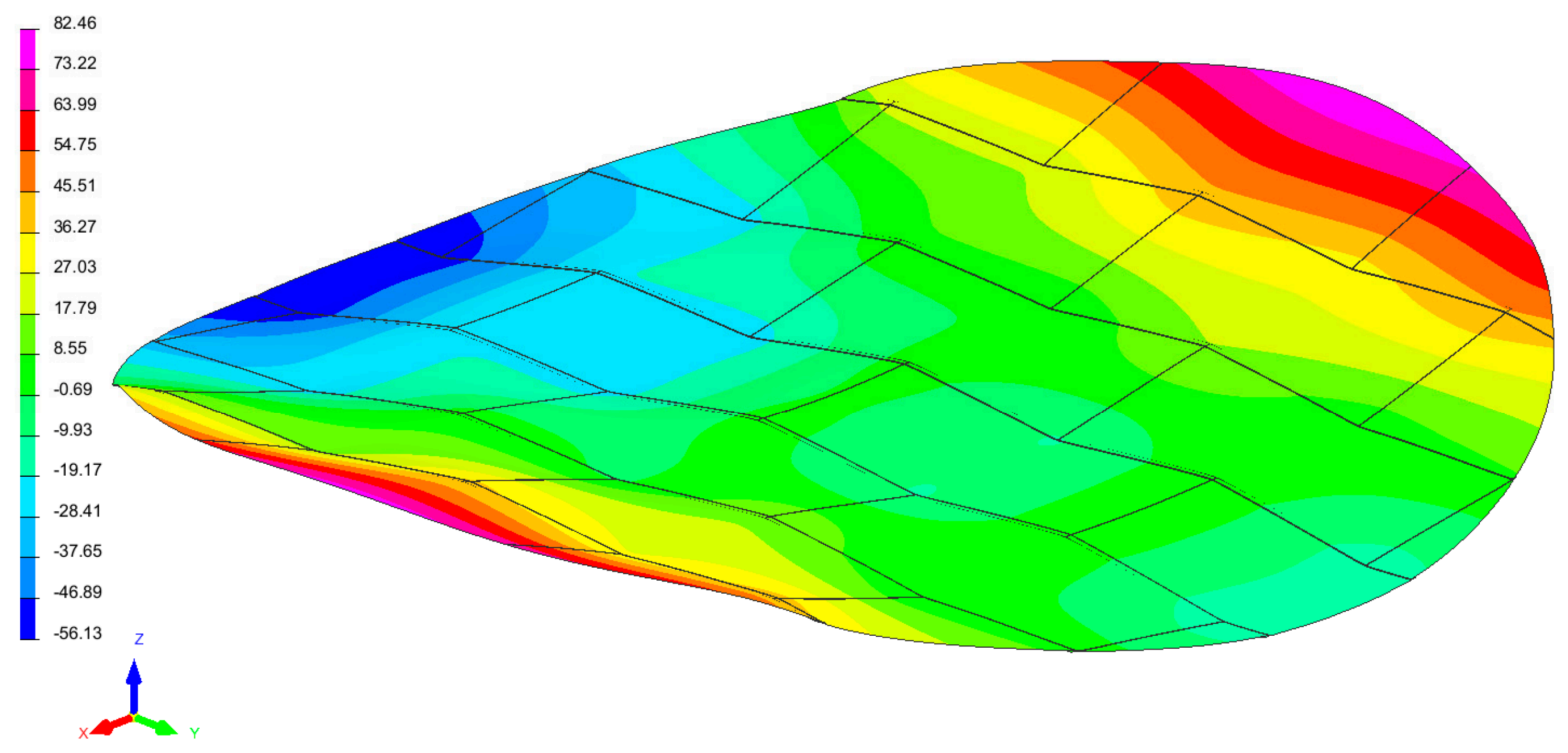
Absolute distortion distribution along the Z axis in the first fixturing variant. Component deformation in mm, scaled 10-fold.

**Figure 20:**
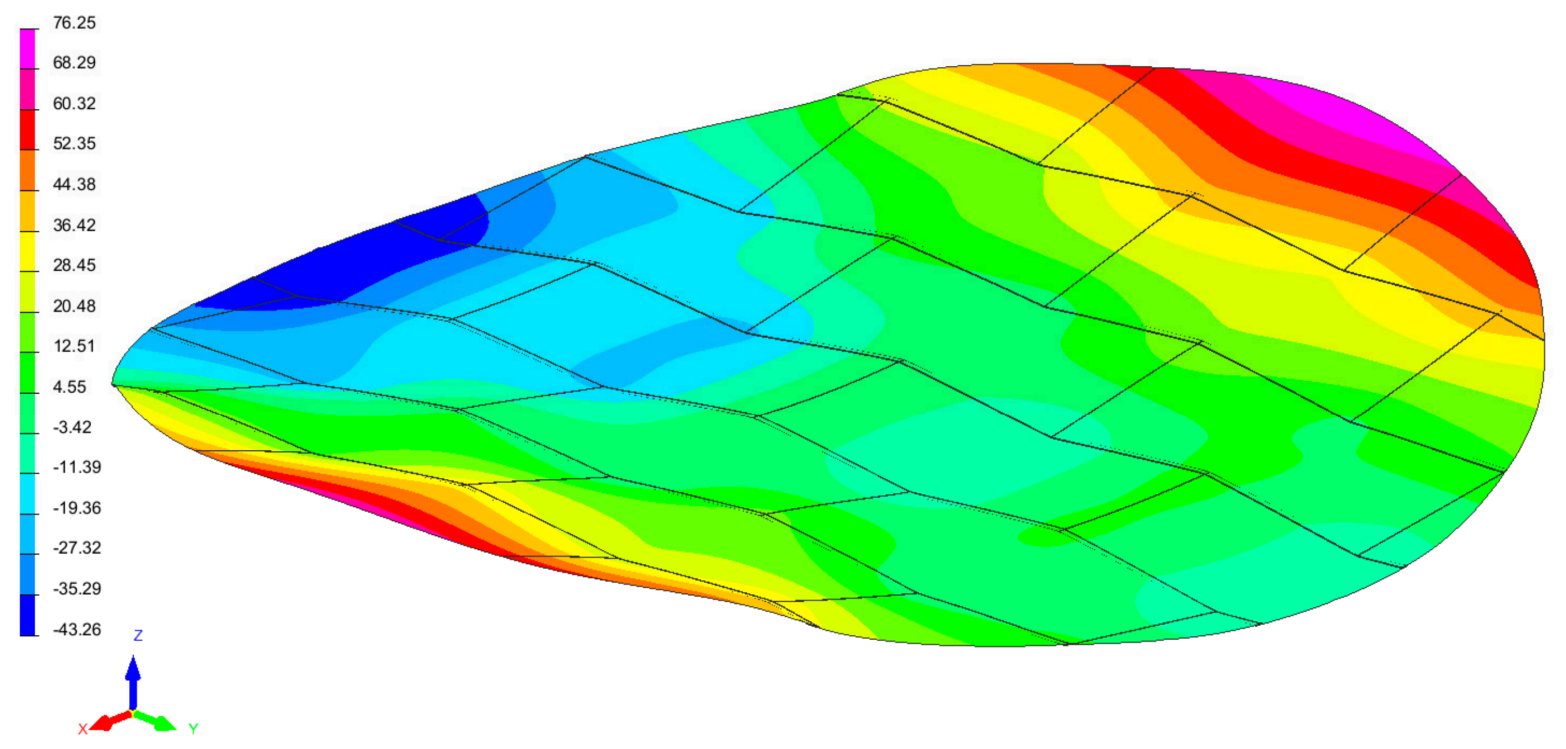
Absolute distortion distribution along the Z axis in the second fixturing variant. Component deformation in mm, scaled 10-fold.

The absolute displacement distributions of the storage tank bottom regions along the X, Y, and Z axes after fixture release are shown in Figure 15, Figure 16, Figure 17, Figure 18, Figure 19 and Figure 20. For both fixturing configurations, the highest sum of absolute displacements in the +X and –X directions was observed in the first variant (Figure 15). The difference between the distortion values in fixturing variants 1 and 2 is 1%. The maximum sum of absolute displacements in the +Y and –Y directions was also found in the first fixturing variant (Figure 17). The difference in distortion values between models 1 and 2 likewise amounts to 1%.

The largest distortion magnitudes among all analyzed directions were observed along the Z axis, perpendicular to the surface of the tank bottom. Displacements along the Z axis were approximately 10-fold higher than those along the X and Y axes. The occurrence of distortions in both the –Z and +Z directions was established. Upon completion of the welding process, the tank bottom assumed the shape of a hyperbolic paraboloid—a quadric surface of negative Gaussian curvature characterized by opposing signs of curvature in perpendicular directions. The maximum sum of absolute displacements in the +Z and –Z directions was observed in the first fixturing variant (Figure 19). The difference between the maximum distortion values in the first fixturing variant and the minimum values in the second fixturing variant is 16%.

**Figure 21:**
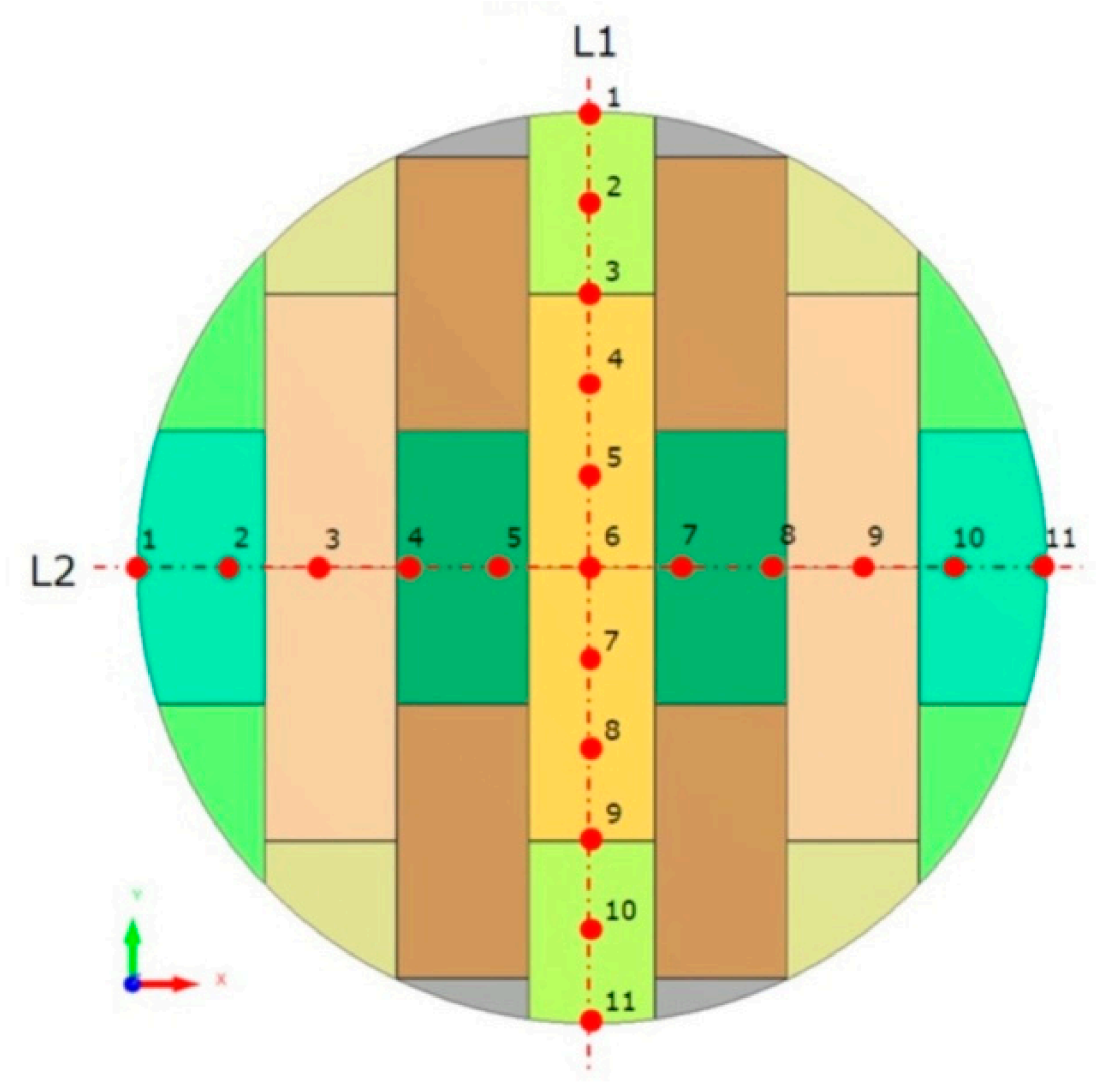
Arrangement schematic of points (lines 1 and 2) for comparative analysis.

For a comparative analysis of the calculated distortions across specific regions of the tank bottom, distortion values at selected points were extracted from the complete dataset (Figure 21). In accordance with the schematic in Figure 19, two measurement lines (L1 and L2) were defined, each consisting of 11 points spaced 1000 mm apart.

The distortion values along the X, Y, and Z axes at the points along measurement lines 1 and 2 for the two tank bottom stiffening variants are presented in Table 1, Table 2 and Table 3. In the tank bottom welding simulation utilizing both variants of the fixture system, no significant differences were observed in the distortion values along the X axis at the points on lines 1 and 2. These distortion magnitudes remain at a comparable level. Likewise, no differences between lines 1 and 2 were found regarding the displacements along the Y axis. The sum and mean of the absolute values in the first fixturing variant were 1% greater than the corresponding sum for the second fixturing variant.

In the case of distortions along the Z axis on line L1, the mean of the absolute values in the first fixturing variant was 21% greater than the mean of the absolute values for the second fixturing variant, and 9% greater on line L2. The sum of the absolute distortion values on line L1 in the first fixturing variant was 21% greater than the sum for the second fixturing variant. The magnitude of these distortions for the points on line 2 was likewise higher in the first fixturing variant by 9%.

### 2.2. Physical Simulation

#### 2.2.1. Physical Simulation Methodology

Based on the numerical simulation results, the second variant of the fixture (tank stiffening) system was selected for the physical simulation of the large-scale storage tank bottom welding process. The analysis of the reservoir bottom deformation distribution following the physical simulation was conducted along the *Z*-axis at the same measurement points as those used in the numerical simulation. Deformations in the X- and *Y*-axis directions were not analyzed in the physical simulation.

**Figure 22:**
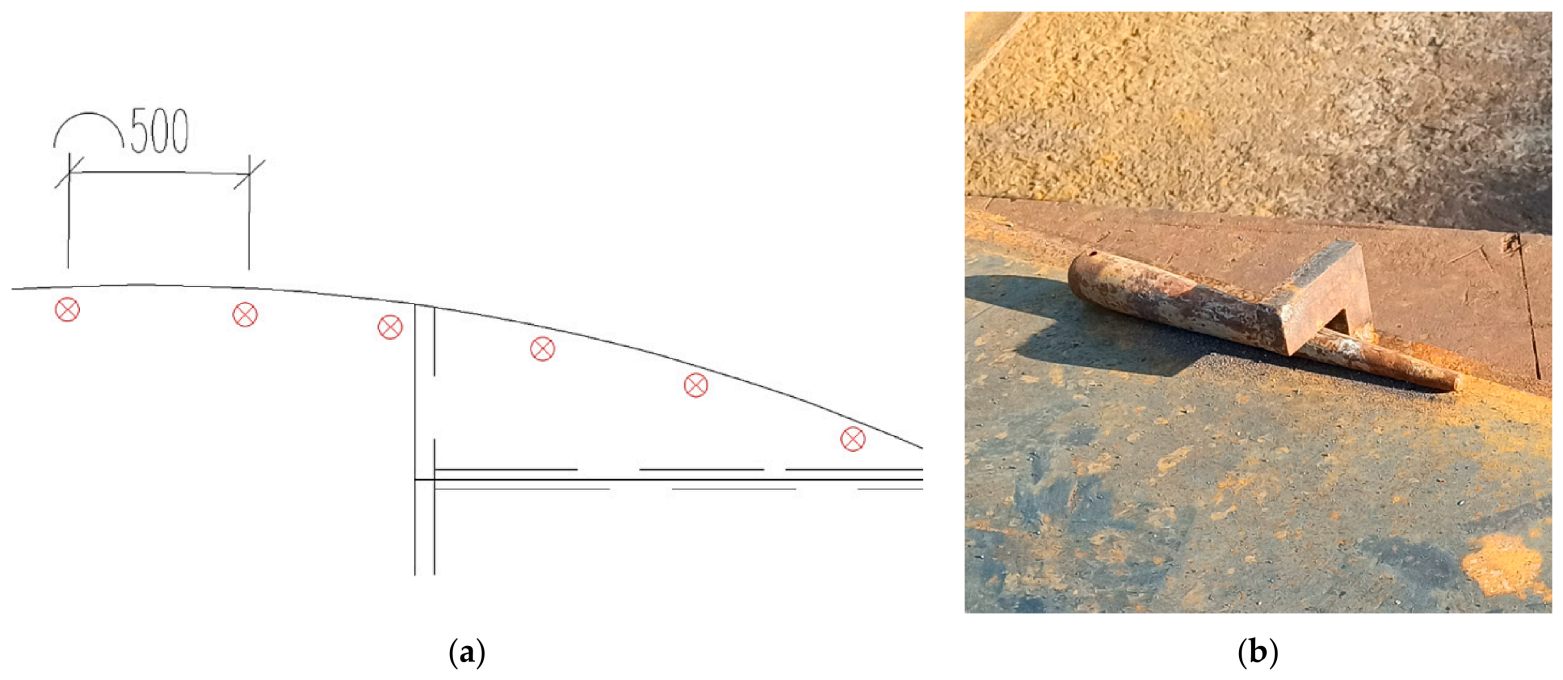
Schematic (**a**) and view (**b**) of the fixture system implemented during the welding of the storage tank bottom. The symbol “x in a circle” indicates the (wedge) restraint system, the view of which is shown in Figure 22b.

The physical simulation of the storage tank bottom welding process involved executing the butt welds first, followed by the fillet welds. The schematic configuration and a photograph of the implemented fixture system (used to stiffen the tank bottom during welding) are presented in Figure 22.

The welding process initiated with plate preparation, after which the components were joined via tack welds. The technological parameters for depositing the tack welds using the MAG method are presented in Table 4.

**Figure 23:**
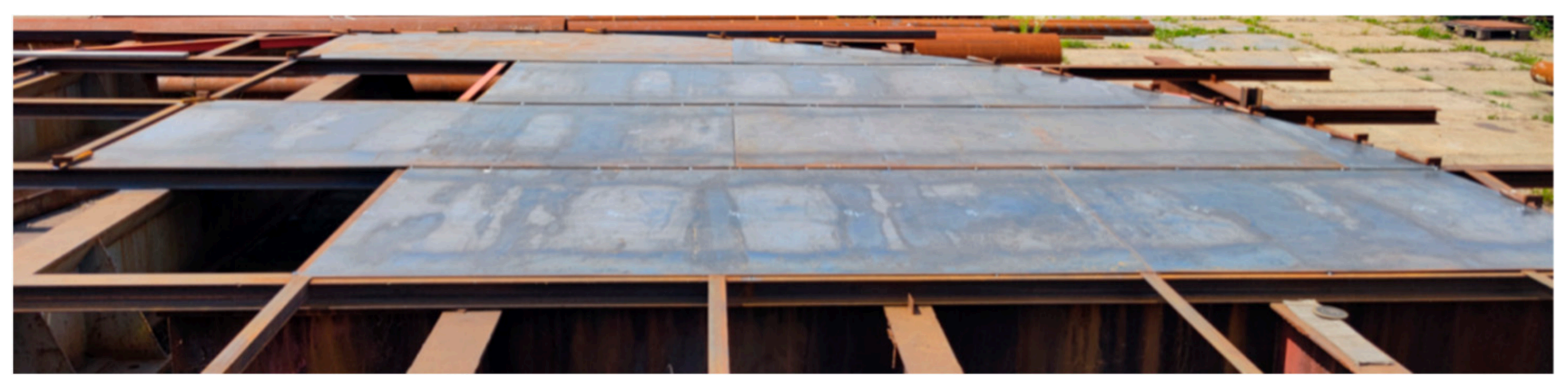
Representative view of the tack welding process history.

Owing to the diameter of the tank bottom, the deposition of the tack welds, butt welds, and fillet welds was performed on an assembly yard utilizing a specially engineered support structure (Figure 23).

Prior to the deposition of the butt welds, distinctive measurement points were marked on the tack-welded plate elements of the tank bottom, and initial baseline measurements were conducted. These measurements involved determining intermediate dimensions (heights) to establish a baseline for quantifying the distortion magnitudes along the Z axis, perpendicular to the tank bottom surface. These dimensions were determined using a cross-line laser emitting two light beams (one vertical and one horizontal), which enabled the quantification of the height differences along the Z axis at the measurement points (before and after welding).

The technical specifications of the cross-line laser are as follows: laser diode: 500–540 nm, <10 mW; working range: 30 m (radius); measurement accuracy: ±0.2 mm at a distance of 1 m; self-leveling range: ±4°. During the measurement procedures, the cross-line laser was positioned at a fixed reference point.

After completing the preliminary measurements, the physical simulation of the tank bottom welding process was carried out. The tank bottom components were welded manually using the MAG welding process. To determine the average welding speed, the welding time for a 30 cm weld bead was measured ten times by an experienced welder who participated in the welding process. Based on these measurements, the average welding speed was determined and subsequently used in the numerical simulation. The butt welds (two passes) were completed first, followed by the fillet welds (two passes). The welding parameters used for the butt and fillet welds are presented in Table 5 and Table 6, respectively.

#### 2.2.2. Physical Simulation Results and Analysis

To determine the welding distortion of the storage tank bottom along the Z axis, the intermediate height values were quantified at the distinctive measurement points (Figure 21). The results of these measurements are presented in Table 7.

**Figure 24:**
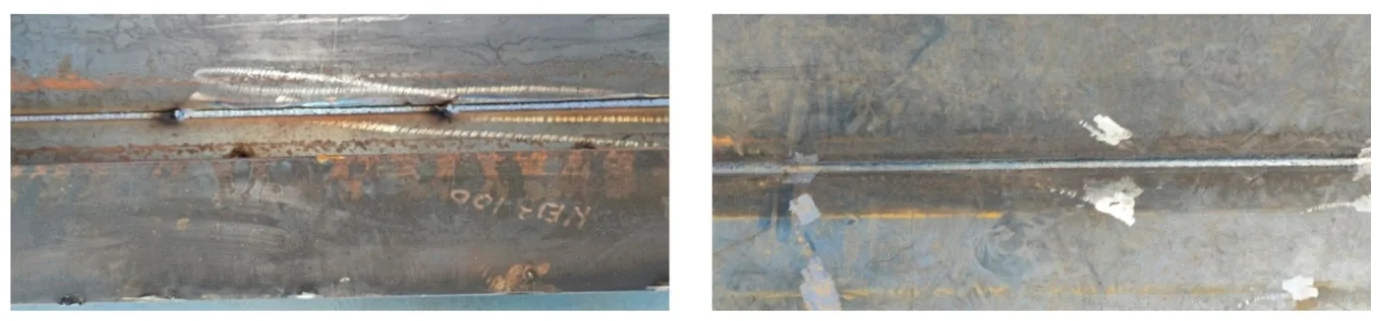
Representative view of the tank bottom butt welds.

**Figure 25:**
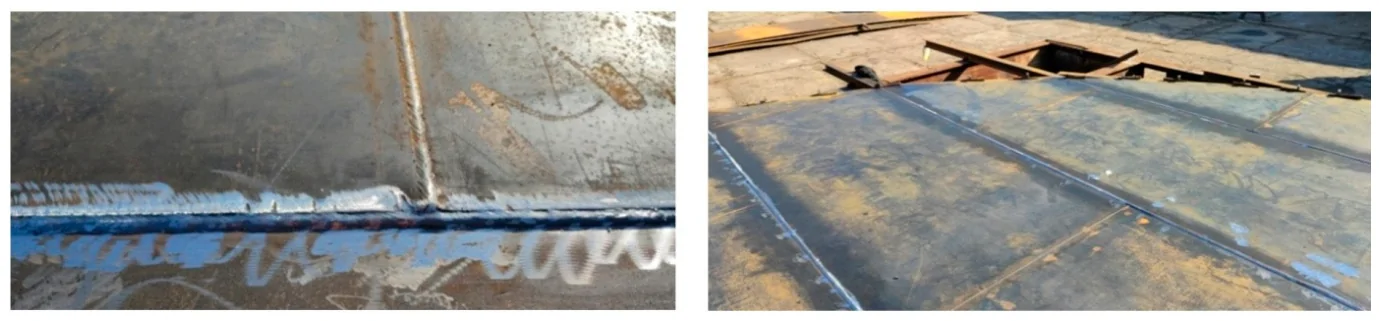
Representative view of the tank bottom butt and fillet welds.

Representative views of the butt and fillet welds on the tank bottom are shown in Figure 24 and Figure 25.

**Figure 26:**
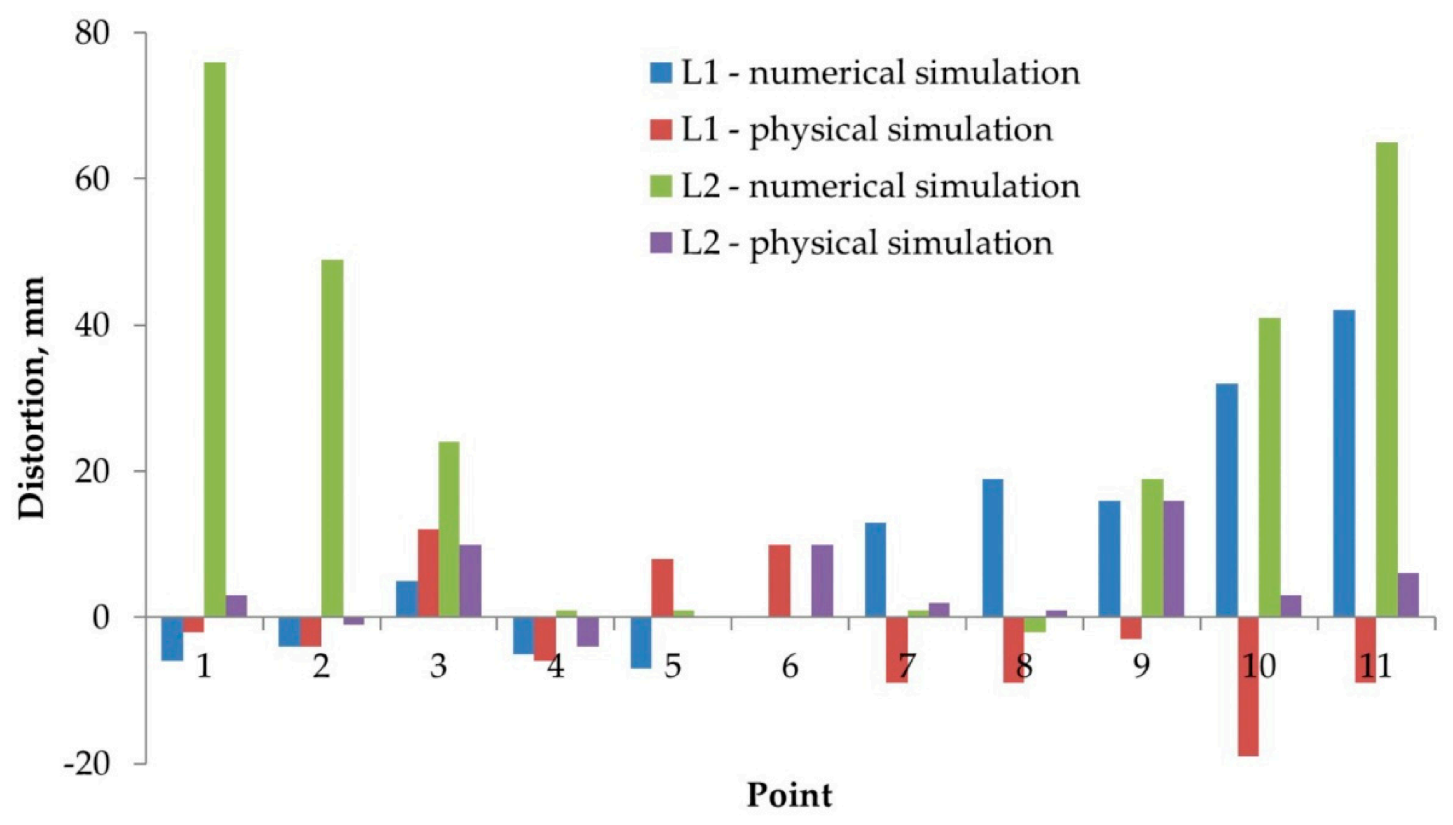
Distortion values along the Z axis at points 1–11 on lines L1 and L2.

The welding distortion values of the storage tank bottom along the Z axis, determined based on the differences in the intermediate dimension values at points 1–11 on lines L1 and L2 relative to the cross-line laser height at the reference point, are presented in Table 8 and Figure 26.

Analysis of the distortions along the Z axis following the physical welding simulation of the large-scale storage tank bottom demonstrated that the distortion values range from −19 to +12 mm for line L1, and from −4 to +16 mm for line L2. Compared to the distortion magnitudes determined via the numerical simulation, the distortion values obtained after the physical simulation are significantly lower at multiple points along lines L1 and L2.

Analyzing the obtained welding distortion values of the storage tank bottom following the physical simulation of the welding process with the second fixture system variant regarding compliance with the 0.2% × D flatness tolerance, it must be stated that distortion values falling within the target tolerance zone of ΔT = 20 mm occur at the measurement points located on line 2. For the majority of the points on line L1, this condition is not satisfied. This applies primarily to points 3 and 6. At points 2, 4, and 5, the absolute deformation values are comparable. At the remaining points, the deformation values obtained from the physical experiment are significantly lower than those predicted by the numerical simulation. Nonetheless, it should be noted that the mean of the absolute distortion values of the storage tank bottom determined after the physical welding simulation with the second fixture system variant is significantly lower compared to the numerical simulation—by over 40% for the points on line L1 and by 80% for the points on line L2.

A comparison of the results obtained via the finite element method (FEM) with the experimental measurement results reveals discrepancies in the quantitative magnitudes of the welding distortions. However, it must be emphasized that such differences are commonly reported in papers focusing on the numerical simulation of welding processes and do not conclusively indicate errors in the adopted model assumptions. The discrepancies stem primarily from the simplifications implemented in the numerical model. Specifically, the utilized material model does not fully account for the complex temperature-dependent behavior, including phase transformations and the associated changes in mechanical properties. These phenomena exert a significant influence on the development of residual stresses and distortions during the welding process.

Another critical factor is the prescription of the boundary conditions. In actual welding processes, the stiffness and the clamping method of the components, as well as the contact conditions between them, are highly difficult to accurately replicate in a numerical model. Even minor variations in the clamping conditions can lead to substantial shifts in distortion levels.

Additionally, the accuracy of the results depends heavily on the adopted heat source model. In the actual welding process, the energy input is non-homogeneous and transient, whereas numerical models utilize simplified and idealized heat source distributions. This directly affects the calculated temperature field and, consequently, the distribution of distortions and stresses.

Despite the indicated limitations, the numerical model allows for a qualitative assessment of the welded structure’s behavior regarding spatial distribution, directions, and relative magnitudes of distortion, which correlate with experimental observations at selected points. This implies that the model provides a reliable representation of the physical mechanisms governing the welding process, despite the lack of full quantitative correlation. Such a level of agreement can be considered sufficient from an engineering standpoint, particularly for large-scale welded structures, where achieving complete numerical correlation is hindered by the complexity of the process.

The identified discrepancies and modeling limitations highlight the challenges associated with the precise simulation of welding processes using FEM. The primary difficulties include high computational costs, high sensitivity of the results to input data, and challenges in accurately replicating process conditions. Consequently, from an engineering application perspective, developing and implementing simplified methods to predict welding distortion is highly justified. Techniques such as the applied shrinkage method provide an efficient alternative, enabling the estimation of distortion fields with significantly lower computational overhead while maintaining sufficient accuracy for practical applications. Achieving full quantitative correlation between numerical simulation results and experimental data in welding processes is rarely attainable in practice due to the highly non-linear and coupled nature of thermo-mechanical phenomena.

## 3. Conclusions

Based on the results of the conducted research, the following conclusions have been formulated:

- The application of two variants of the fixture system to stiffen the components during the welding of the large-scale storage tank bottom resulted in distinct welding distortion magnitudes. Upon completion of the welding process, the distortion fields exhibited variations in value while maintaining a similar geometric character. The tank bottom assumed the shape of a hyperbolic paraboloid—a quadric surface of negative Gaussian curvature characterized by opposing signs of curvature in perpendicular directions.
- Dimensionally significant distortions of the tank bottom occurred along the Z axis, perpendicular to the surface of the tank bottom. The maximum displacements were observed in the peripheral regions of the modeled structure across all analyzed fixturing configurations during welding. However, it should be emphasized that any statements regarding deformations in the *X*- and *Y*-axis directions are based solely on the results of the numerical simulation and, therefore, on data that have not been experimentally validated.
- Analysis revealed that the second variant of the fixture system was characterized by lower resultant distortion magnitudes. Based on the conducted numerical simulations, it was established that a greater number of fixtures (clamps), corresponding to the first variant of the fixture system, can lead to higher distortion levels. However, both variants of the fixture system must be evaluated by considering the clamping force with which the fixtures (wedges) hold the components stationary during welding. If a smaller number of clamps (wedges) and a lower clamping force magnitude are applied, the total clamping force may be insufficient. This will alter the structural constraint condition derived from this force, thereby modifying the welding distortion field.
- The conducted numerical simulation of the tank bottom welding process enabled a qualitative verification of the welding sequences (WPS instructions) from the perspective of welding distortion. The presented results, analyses, and conclusions are qualitative rather than quantitative and must be evaluated within the context of the adopted boundary conditions. The same conditions and simplifications were applied to both fixturing variants, allowing for the determination of the influence of both fixture systems on the welding distortion distribution. Analysis of the obtained results indicated that the second variant of the fixture system should be selected as the subject for further tank bottom distortion analyses during physical simulation.
- Analysis of the welding distortion distribution results across the tank bottom during the physical simulation, regarding compliance with the target bottom plate flatness tolerance (0.2% × D), demonstrated that the second variant of the fixture system requires further refinement. A critical element of future work will involve modeling the clamping force magnitude to achieve a structural constraint condition that guarantees a reduction in welding distortion values to the target level across all regions (points) of the storage tank bottom.

## Figures and Tables

**Table 1:** Distortion values along the X axis at points on lines 1 and 2 (L1 and L2) after releasing the fixtures configured according to variants 1 and 2.

Point	Distortion Values, mm			
	Variant 1		Variant 2	
	L1	L2	L1	L2
1	−0.01	1.26	−0.04	1.25
2	0.00	1.17	0.00	1.16
3	0.00	0.76	0.00	0.76
4	0.00	0.41	0.00	0.41
5	0.00	0.31	0.00	0.31
6	0.00	0.00	0.00	0.00
7	0.00	−0.30	0.00	−0.30
8	0.00	−0.41	0.00	−0.41
9	0.00	−0.75	0.00	−0.74
10	0.00	−1.15	0.00	−1.13
11	0.00	−1.24	0.04	−1.21
Mean of absolute values	0.00	0.71	0.01	0.70
Sum of absolute values	0.01	7.76	0.08	7.68

**Table 2:** Distortion values along the Y axis at points on lines 1 and 2 (L1 and L2) after releasing the fixtures configured according to variants 1 and 2.

Point	Distortion Values, mm			
	Variant 1		Variant 2	
	L1	L2	L1	L2
1	−0.79	0.10	−0.79	0.10
2	−0.72	0.10	−0.73	0.11
3	−0.63	−0.04	−0.63	−0.04
4	−0.21	0.15	−0.22	0.15
5	−0.09	0.15	−0.10	0.15
6	0.00	0.00	0.00	0.00
7	0.44	0.15	0.43	0.15
8	0.56	0.15	0.55	0.15
9	0.98	−0.04	0.97	−0.04
10	1.06	0.10	1.06	0.10
11	1.11	0.10	0.75	0.10
Mean of absolute values	0.60	0.10	0.57	0.10
Sum of absolute values	6.59	1.08	6.23	1.09

**Table 3:** Distortion values along the Z axis at points on lines 1 and 2 (L1 and L2) after releasing the fixtures configured according to variants 1 and 2.

Point	Distortion Values, mm			
	Variant 1		Variant 2	
	L1	L2	L1	L2
1	12.78	81.90	−6.48	75.89
2	−8.27	51.40	−4.41	48.74
3	3.26	25.07	5.19	24.41
4	−5.54	0.21	−4.92	0.60
5	−6.77	1.24	−6.73	1.44
6	−0.04	−0.04	−0.04	−0.04
7	14.59	0.68	13.47	0.72
8	21.89	−1.85	19.13	−2.23
9	21.68	21.62	16.35	18.82
10	41.25	47.71	32.12	41.06
11	55.92	76.88	41.87	64.97
Mean of absolute values	17.45	28.05	13.70	25.36
Sum of absolute values	191.95	308.60	150.71	278.92

**Table 4:** Technological parameters for tack welding.

Parameter	Value
Welding current	215 A
Current type	DC+
Arc voltage	21.8 V
Wire feed speed	7 m/min
Wire type	Gold G3Si1, Ø1.2 mm
Shielding gas	18% CO_2_, 82% Ar
Gas flow rate	28 L/min

**Table 5:** Technological parameters for depositing butt welds on the storage tank bottom plate elements.

Parameter	Value	
	Pass 1	Pass 2
Welding current	185 A	230 A
Current type	DC+	DC+
Arc voltage	18 V	21 V
Wire feed speed	5 m/min	7 m/min
Wire type	Gold G3Si1, Փ1.2 mm	Gold G3Si1, Փ1.2 mm
Shielding gas	18% CO_2_, 82% Ar	18% CO_2_, 82% Ar
Gas flow rate	28 L/min	28 L/min
Welding speed	262 mm/min	270 mm/min

**Table 6:** Technological parameters for depositing fillet welds on the storage tank bottom plate elements.

Parameter	Value	
	Pass 1	Pass 2
Welding current	208 A	220 A
Current type	DC+	DC+
Arc voltage	19.5 V	21.5 V
Wire feed speed	6 m/min	7 m/min
Wire type	Gold G3Si1, Փ1.2 mm	Gold G3Si1, Փ1.2 mm
Shielding gas	18% CO_2_, 82% Ar	18% CO_2_, 82% Ar
Gas flow rate	28 L/min	28 L/min
Welding speed	525 mm/min	290 mm/min

**Table 7:** Intermediate dimension values at points 1–11 on lines L1 and L2 (Figure 21), determined using the cross-line laser.

Point	Intermediate Dimension Value (Height Along the Z Axis), mm	
	Line 1	Line 2
1	2450	2445
2	2452	2449
3	2436	2438
4	2454	2452
5	2440	2448
6	2438	2438
7	2457	2446
8	2457	2447
9	2451	2432
10	2467	2445
11	2457	2442
Height at the reference point: 2448 mm		

**Table 8:** Distortion values at points 1–11 on lines L1 and L2 (Figure 21).

Point	Distortion Values, mm (Rounded to the Nearest 1 mm)			
	Line 1		Line 2	
	Numerical Sim.	Physical Sim.	Numerical sim.	Physical Sim.
1	−6	−2	76	3
2	−4	−4	49	−1
3	5	12	24	10
4	−5	−6	1	−4
5	−7	8	1	0
6	0	10	0	10
7	13	−9	1	2
8	19	−9	−2	1
9	16	−3	19	16
10	32	−19	41	3
11	42	−9	65	6
Mean of absolute values	14	8	25	5
Sum of absolute values	149	91	279	56

## Data Availability

The original contributions presented in this study are included in the article. Further inquiries can be directed to the corresponding author.
